# Characterization of Inflammation in Delayed Cortical Transplantation

**DOI:** 10.3389/fnmol.2019.00160

**Published:** 2019-06-25

**Authors:** Nissrine Ballout, Tristan Rochelle, Sebastien Brot, Marie-Laure Bonnet, Maureen Francheteau, Laetitia Prestoz, Kazem Zibara, Afsaneh Gaillard

**Affiliations:** ^1^Laboratoire de Neurosciences Expérimentales et Cliniques, Université de Poitiers, INSERM U1084, Poitiers, France; ^2^Laboratory of Stem Cells, PRASE, DSST, Department of Biology, Faculty of Sciences-I, Lebanese University, Beirut, Lebanon; ^3^CHU Poitiers, Poitiers, France

**Keywords:** motor cortex, cortical lesion, embryonic transplantation, neuroinflammation, delay

## Abstract

We previously reported that embryonic motor cortical neurons transplanted 1-week after lesion in the adult mouse motor cortex significantly enhances graft vascularization, survival, and proliferation of grafted cells, the density of projections developed by grafted neurons and improves functional repair and recovery. The purpose of the present study is to understand the extent to which post-traumatic inflammation following cortical lesion could influence the survival of grafted neurons and the development of their projections to target brain regions and conversely how transplanted cells can modulate host inflammation. For this, embryonic motor cortical tissue was grafted either immediately or with a 1-week delay into the lesioned motor cortex of adult mice. Immunohistochemistry (IHC) analysis was performed to determine the density and cell morphology of resident and peripheral infiltrating immune cells. Then, *in situ* hybridization (ISH) was performed to analyze the distribution and temporal mRNA expression pattern of pro-inflammatory or anti-inflammatory cytokines following cortical lesion. In parallel, we analyzed the protein expression of both M1- and M2-associated markers to study the M1/M2 balance switch. We have shown that 1-week after the lesion, the number of astrocytes, microglia, oligodendrocytes, and CD45+ cells were significantly increased along with characteristics of M2 microglia phenotype. Interestingly, the majority of microglia co-expressed transforming growth factor-β1 (TGF-β1), an anti-inflammatory cytokine, supporting the hypothesis that microglial activation is also neuroprotective. Our results suggest that the modulation of post-traumatic inflammation 1-week after cortical lesion might be implicated in the improvement of graft vascularization, survival, and density of projections developed by grafted neurons.

## Introduction

Loss of cortical neurons is a common characteristic of numerous neuropathological conditions. The inhibitory nature of the adult mammalian central nervous system (CNS) prevents spontaneous axonal regeneration following injury (Davies et al., 1997, 1999), which could be overcome by transplantation of embryonic neurons. We have previously reported that embryonic motor cortical neurons grafted immediately after a cortical lesion of the adult mouse motor cortex allowed reestablishment of the damaged motor pathways. Indeed, the transplanted neurons developed projections towards all cortical and subcortical targets of the motor cortex, including distant targets such as the spinal cord (Gaillard et al., 2007; Ballout et al., 2016). While these results were encouraging for CNS repair, a serious limitation in a clinical setting is the time delay of transplantation after injury as neurons derived from embryonic or induced pluripotent stem cells may not be immediately available in terms of harvesting, cell processing, and transplantation (Cox et al., 2017; Wang et al., 2017). We have recently shown that a 1-week delay between the cortical lesion and transplantation can significantly enhance graft vascularization, cell proliferation, survival, and density of projections developed by grafted neurons, leading to a beneficial impact on functional repair and recovery (Péron et al., 2017). However, mechanisms responsible for this improvement attributed to delay are not well-defined. It could be hypothesized that potential benefits may be due to the release of trophic factors secreted by cells surrounding the lesion (Nieto-Sampedro et al., 1983), the secretion of pro-angiogenic factors (Sköld et al., 2005; Dray et al., 2009), or a decrease in toxin (Gonzalez and Sharp, 1987) and inflammation levels, characterized by activated microglia and astrocytes (Zhang et al., 2010; Burda et al., 2016).

The response of astrocytes to injury proceeds through several stages and depends on the extent of trauma. Within 24 h after injury, there is a rapid activation of astrocytes whose main function is to create a physical barrier between damaged and healthy tissue (Raivich et al., 1999). Activation of astrocytes may have antagonistic effects depending on the context in which they occur, i.e., the degree, type and time point after injury (Farina et al., 2007). For example, the production of neurotrophic factors by astrocytes and reduction in the spread of toxic substances released from dead cells promote neuronal survival (Faulkner et al., 2004; Myer et al., 2006; Rolls et al., 2009; Sofroniew and Vinters, 2010). Conversely, glial scar formation impairs adult CNS regeneration (Itoh et al., 2007; Wanner et al., 2008; Rolls et al., 2009). In parallel, released pro-inflammatory cytokines, such as tumor necrosis factor α (TNFα), inhibit neurite growth and kill oligodendrocytes (Neumann et al., 2002).

Phagocytic immune cells found around the lesion site are mainly composed of two types: specialized CNS-resident microglia and infiltrating macrophages. Microglia are active contributors to neuronal damage in neurodegenerative diseases (Block et al., 2007), whose response in acute injury reaches its maximum at 5–7 days after injury (Davalos et al., 2005; Ladeby et al., 2005), before gradually disappearing. As for macrophages, they infiltrate into the brain as early as 12 h post-injury and recruit more neutrophils by releasing pro-inflammatory cytokines and up-regulating adhesion molecules in endothelial cells (Huang et al., 2006; Gelderblom et al., 2009). After activation, microglial cells and macrophages proliferate and migrate to the site of injury where they can have two phenotypes (Gordon, 2003; Mantovani et al., 2004). Depending on the stimuli in their local microenvironment, they can be polarized to have distinct effector functions (Colton, 2009; Xiong et al., 2016). Studies have shown that the presence of lipopolysaccharides (LPS) and TNFα promote M1 phenotype (Kumar et al., 2013). The latter produces high levels of pro-inflammatory cytokines such as interleukin 1 and 6 (IL-1 and IL-6), leukemia inhibitory factor (LIF; Chao et al., 1995) and oxidative metabolites that are essential for host defense and phagocytic activity, but that can also cause damage to healthy cells and tissues (Lynch, 2009). In contrast, activated microglia/macrophages, in the presence of anti-inflammatory cytokines such as IL-4 and IL-10, promote M2 phenotype (Chhor et al., 2013) and reduce M1 cytokines and other pro-inflammatory mediators (Gordon, 2003; Mantovani et al., 2004). It is thought that M2 microglia/macrophages promote repair processes such as angiogenesis by secreting anti-inflammatory cytokines such as transforming growth factor-β1 (TGF-β1; Kiefer et al., 1996). Moreover, they may enhance neuronal survival by removing cell debris (Rapalino et al., 1998) and releasing protective neurotrophic factors such as nerve growth factor (NGF), brain-derived neurotrophic factor (BDNF) or glial cell-line derived neurotrophic factor (GDNF; Neumann et al., 2006; Schwartz et al., 2006; Madinier et al., 2009). Furthermore, they can also stimulate axonal regeneration and enhance the turnover and maturation of oligodendrocyte lineage cells (Schonberg et al., 2007; Gensel et al., 2008).

Several clinical studies showed that transplantation of stem cells is feasible, safe and therapeutically promising. For instance, autologous bone marrow mononuclear cells (BM-MNCs) have been injected intravenously within 36–48 h of injury to treat traumatic brain injury (TBI) patients (Cox et al., 2017). Post-treatment follow-up showed a trend for higher preservation of the white matter volume and a reduction in pro-inflammatory cytokines. Another clinical study investigated the feasibility and safety of intravenous or intrathecal injections of neural stem cells from BM-MNCs at 20–60 days after severe TBI (Wang et al., 2017). For 6 months, no major complications were observed, the neurological score of seven patients over 10 was improved and serum levels of neurotrophic factors were higher following transplantation.

Currently, no pharmacological treatment has received FDA approval for patients with TBI (Diaz-Arrastia et al., 2014). One of the reasons for the absence of treatment is that most preclinical studies measure drugs directly or soon after TBI (Diaz-Arrastia et al., 2014). This experimental protocol does not take into consideration the treatment gap observed in clinical cases after brain trauma (Tanielian and Jaycox, 2008; Demakis and Rimland, 2010). Some drugs limit considerably the extent of secondary injury, they are effective by targeting one mechanism of secondary injury. The progress of secondary injury induces the loss of drug targets which reduces rapidly their efficacy and their therapeutic effect of targeting one mechanism of secondary injury. Thus, drugs delivered at longer intervals after injury may have multiple targets which can still reduce secondary injury. As for stem cells, the therapeutic window remains unclear, whereas stem cells can be used for neuroprotective strategy, for cell replacement strategy or both.

Taken together, the impact of inflammatory responses on neuronal survival seems to depend on a balance between pro- and anti-inflammatory mechanisms induced by different mediators (Bernardino et al., 2005; Vezzani et al., 2008). Inflammatory changes occurring in the post-lesioned environment and their effects on axonal regeneration in adult CNS have been extensively investigated. However, few studies examined the deleterious or beneficial consequences of these changes on axonal growth of transplanted embryonic neurons in the injured adult brain. Therefore, in the present study, we aimed at characterizing the impact of a 1-week delay between the motor cortical lesion and transplantation on post-traumatic inflammation. For this, we have characterized the density, morphology, and phenotype of resident and peripheral infiltrating immune cells, the distribution and temporal mRNA expression pattern of pro- and anti-inflammatory cytokines and the temporal kinetics of microglia/macrophage polarization.

## Materials and Methods

### Animals

**Figure 1:**
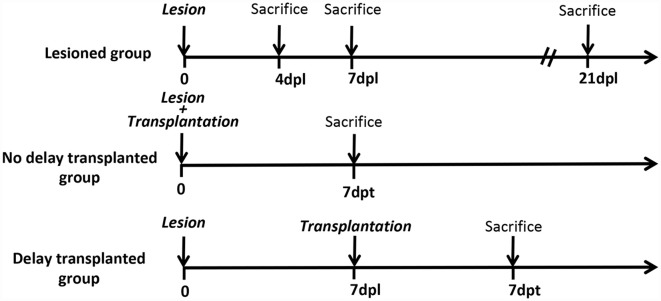
Timeline of the study. Timeline of the study representing the different groups and the different time points of mice sacrifices. Dpl, days post-lesion; dpt, days post-transplantation.

All animal experimentation and housing were carried out in accordance with the guidelines of the French Agriculture and Forestry Ministry (decree 87849) and the European Communities Council Directive (2010/63/EU). The procedures referenced under the file number APAFIS#4928–20 16041 117503028 v3, were approved by ethics committee N°84 COMETHEA Poitou-Charentes. All experiments were conducted in compliance with current Good Clinical Practice standards and in accordance with relevant guidelines and regulations and the principles set forth under the Declaration of Helsinki (1989). All efforts were made to reduce the number of animals used and their suffering. A total of 82 C57BL/6 mice were used in this study: 16 mice were used as controls (without a lesion), 66 mice were lesioned, among which 42 mice were used as “lesioned group” (without transplantation) 12 mice were transplanted without delay (immediately after lesion) and 12 mice were transplanted with 1-week delay (Figure 1).

### Lesion and Transplantation Procedures

Adult (4–6 months old) C57BL/6 mice (*n* = 66, Janvier Labs, Le Genest-Saint-Isl, France) were lesioned. Briefly, animals were anesthetized with a mixture of xylazine/ketamine (intra-peritoneal, ip., 10 and 100 mg/kg, respectively) and the motor cortex was aspirated from 0.5 to 2.5 mm rostral to the Bregma and from 0.5 to 2.5 mm lateral to the midline, with the corpus callosum left intact. Among these mice, 42 were used in the “lesioned group” and 24 were transplanted as described previously (Gaillard et al., 1998, 2007). The transplanted mice were selected randomly. Motor cortical tissue was obtained from embryonic day 14 transgenic mice overexpressing the enhanced green fluorescent protein (EGFP) under the control of a chicken β-actin promotor [C57BL/6-TgN(beta-act-EGFP)] Osb strain (Okabe et al., 1997). Motor cortical tissue was deposited into the host lesion cavity either immediately, without delay (*n* = 12), or with a delay of 1-week (*n* = 12) after the lesion. Care was taken to maintain the original dorso-ventral and anteroposterior orientations of the cortical fragments during the transplantation procedure. We did not perform immunosuppression during transplantation since it has been demonstrated in several previous studies including ours (Gaillard et al., 2007, 2009; Thompson et al., 2009; Klein et al., 2013; Wang et al., 2016; Péron et al., 2017), that immunosuppression is not necessary for grafted fetal mouse cells to survive in a mouse brain as performed in the present study. No animal was excluded after histological analysis.

### Tissue Processing and Immunohistochemistry (IHC)

At different time points (Figure 1), mice were injected with a lethal dose of xylazine/ketamine and perfused transcardiacally with 100 ml of saline (0.9%), followed by 200 ml of ice-cold paraformaldehyde (PFA, 4%) in 0.1 M phosphate buffer (PB, pH 7.4). Brains were removed, post-fixed in 4% PFA overnight at 4°C, and cryoprotected in 30% (w/v) sucrose, 0.1 M sodium phosphate buffer (pH 7.4). Brains were cut in six series on a freezing microtome (Microm HM450, Thermo Scientific) in 40 μm-thick coronal sections and stored in a cryoprotective solution (20% glucose, 40% ethylene glycol, 0.025% sodium azide, 0.05M phosphate buffer pH 7.4). For immunohistochemistry (IHC), free-floating sections were incubated in a blocking solution [3% bovine serum, 0.3% Triton X-100 in phosphate-buffered saline (PBS) 0.1 M pH 7.4] for 90 min at room temperature (RT). Primary antibodies, diluted in blocking solution, were applied overnight at 4°C. Appropriate secondary antibodies were diluted in blocking solution and applied for 1 h at RT. The following antibodies were used to label activated microglia and hematopoietic cells, astrocytes, oligodendrocytes and neurons, respectively: rabbit anti-Iba1 (1:500, Wako) and rat anti-CD45 (1:500, Abcam), chicken anti-Glial fibrillary acidic protein (GFAP; 1:1,000, Abcam), rabbit anti-olig2 (1:500, Millipore) and mouse anti-NeuN (1:500, Millipore). Rabbit anti-CD86 (1:200, Abcam) and goat anti-Arg1 (1:250, Santa Cruz) were used for M1 and M2 phenotype respectively. Rat anti-C3 (1:200, Abcam) and rabbit anti-CD109 (1:200, Abcam) were used for A1 and A2 phenotype respectively. Chicken anti-green fluorescent protein (GFP; 1:1,000, Abcam) or Rabbit anti-GFP (1:1,000, Invitrogen) were used to label transplanted cells whereas nuclei were labeled with DAPI (1:2,000, Sigma). The sections were covered with DePeX (VWR) mounting medium.

### *In situ* Hybridization (ISH)

For *in situ* hybridization (ISH), brains were collected, cryoprotected in 30% (w/v) sucrose and quickly frozen in isopentane (2-methylbutane, VWR) cooled at −45°C. Brains were then cut in 6 series on a cryostat (HM550, Microm) in 16 μm-thick coronal sections, mounted on super-frost slides (Superfrost Plus, VWR) and stored at −80°C.

ISH was performed in order to characterize the spatiotemporal expression of pro-inflammatory cytokines IL-1α, IL-1β, IL-6, TNFα, and LIF and anti-inflammatory cytokines: TGFβ1, IL-4, and IL-10, at day 0, 4 and 7 days after cortical lesion. Specific digoxygenin-labeled cRNA probes were prepared from cDNA fragments (450 bp to 800 bp) of these murine cytokines. cDNAs were amplified by polymerase chain reaction (PCR) using specific primers from cDNA banks obtained from different sources (brain, liver, spleen, skin, adipose tissue and LPS-treated bone marrow-derived macrophages). cDNA fragments were then cloned into pGEM^®^-T Easy vectors (Promega, Charbonnières-les-Bains, France) and verified by sequencing. Complementary (antisense) and non-complementary (sense) RNA probes were produced using T7 or SP6 RNA polymerase (Riboprobes^®^ System-T7, Promega Corporation, Madison, WI, USA). Before exposing to the probes, sections were digested by proteinase K (5 μg/ml) for 10 min at 37°C followed by an acetylation step in triethanolamine buffer (100 mM triethanolamine, 0.25% acetic anhydride) to reduce non-specific binding. Hybridization was carried out overnight at 65°C in a humidified chamber using probes at a final concentration of 500 ng/ml diluted in hybridization buffer containing 50% formamide, 1× Denhardt’s solution, 10% dextran sulfate, 1 mg/ml yeast tRNA in salt solution (200 mM NaCl, 10 mM Tris-HCl pH 7.5, 10 mM phosphate buffer pH 7.4, 5 mM EDTA pH 8). The following day, sections were washed at RT in 1X sodium saline citrate (SSC), 50% formamide, 0.1% Tween 20 at 65°C, and in MABT buffer (0.15 M NaCl, 0.1 M Maleic acid, 0.2 M NaOH, 0.1% Tween 20, pH 7.5). After blocking in 10% B10 reagent (Roche Diagnostics, Mannheim, Germany) and 10% sheep serum, sections were incubated overnight at RT with an alkaline phosphatase-labeled anti-digoxigenin antibody (Roche Diagnostics, Mannheim, Germany) diluted 1:2,000 in blocking buffer. Sections were finally washed with NTMT buffer (0.1 M NaCl, 0.1 M Tris–HCl pH 9.5, 0.05 M MgCl_2_, 0.1 M Tween 20, pH 9.5) before being incubated in detection buffer containing 0.045% nitroblue tetrazolium, 0.35% 5-bromo-4-chloro-3-idolyl phosphate (Roche Diagnostics, Mannheim, Germany) and 0.1% levamisole (Sigma) in NTMT buffer. Slides were then dried and mounted with DePeX (BDH Laboratories, Poole, UK). Results with antisense probes were compared with sense probes and were confirmed by testing six animals for each group.

The anti-Iba1 antibody was used to define whether macrophage/microglia could be the source of IL-1β and TGF-β1. Briefly, after ISH, sections were incubated in blocking solution (3% bovine serum, 0.3% Triton X-100 in PBS 0.1 M pH 7.4) for 90 min at RT. Anti-Iba1 primary antibody (1:500, Wako) diluted in blocking solution was applied overnight at 4°C. Sections were then incubated with biotinylated goat anti-rabbit antibody (1:200, Vector, Burlingame, CA, USA) for 1 h 30 min at RT. After washing, sections were treated with 0.3% hydrogen peroxide (Sigma, Seelze, Germany) to quench endogenous peroxidases and were reacted with avidin-biotin peroxidase complex (Vectastain^®^ ABC Kit, Vector, Burlingame, CA, USA) for 1 h at RT. The sections were subsequently incubated in 0.1 M PB containing 0.33 mg/ml 3,3′-diaminobenzidine tetrahydrochloride (Sigma, St Louis, MO, USA) and 0.0006% hydrogen peroxide. Mounted sections were dried and covered with DePeX (VWR).

### Western Blot Analysis

At different time points after the lesion (Figure 1), mice were injected with a lethal dose of xylazine/ketamine and perfused transcardiacally with 100 ml of saline (0.9%), then brains were removed and kept on ice. The control (intact animal) and lesioned cortex were collected and sonicated using protein lysis buffer (RIPA 50 mM Tris pH 8.0, 150 mM NaCl, 1% NP-40, 0.1% SDS, 0.5% sodium deoxycholate with protease cocktail inhibitor). The brain homogenates were then incubated for 2 h at 4°C, centrifuged at 13,000 rpm for 5 min at 4°C, and the supernatants were then collected and stored at −80°C. After protein extraction, equal amounts of proteins were loaded either onto 7.5% or 10% resolving gel for electrophoresis then transferred onto a nitrocellulose membrane (Bio-Rad, Munich, Germany). Membranes were blocked with 5% milk powder in PBS 0.1 M, 0.1% Tween 20 and incubated overnight at 4°C with primary antibodies. The following antibodies were used: rabbit anti-CD86 (M1 phenotype, 1/1,000, Abcam), goat anti-CD206 (M2 phenotype, 1/500, R&D systems, Minneapolis, MN, USA), goat anti-Arg1 (M2 phenotype, 1/250, Santa Cruz, CA, USA), and mouse anti-α tubulin (Loading control, 1/2,000, Sigma). After washing three times using 0.1 M PBS, 0.1% Tween 20, the membrane was incubated with horseradish peroxidase (HRP)-conjugated anti-rabbit, anti-goat or anti-mouse immunoglobulin G (1/50,000, Jackson ImmunoResearch, West Grove, PA, USA) for 1 h at RT. The membranes were washed three times and then revealed by Luminata Forte Western HRP substrate (Millipore, MA, USA). Pictures were taken using the PXi imaging system (Syngene, Cambridge, UK) and band intensity was quantified using ImageJ software (Bethesda, MD, USA).

### Data Acquisition and Quantification

For each mouse, images of injury area were acquired with a Zeiss Axio Imager. M2 Apotome microscope at x20 magnification, at the rostral, middle and caudal part of the lesion or the graft. On mosaic acquisition, three images corresponding to the areas of interest were used for all quantifications using ZEN software (Zeiss). For control mice, equivalent sections were selected at the same anteroposterior coordinates as the lesioned sections. Areas of interest were further analyzed and photographed with a confocal laser-scanning microscope FV1000 (Olympus, Rungis, France). The counts were expressed per mm^2^.

### Statistical Analysis

Statistical analyses were performed using a two-tailed student’s *t*-test, two-way analysis of variance (ANOVA) followed by a Bonferroni correction or Kruskal-Wallis test followed by Dunn’s multiple comparisons test. Data are expressed as mean ± SEM. Differences were considered statistically significant when *p* < 0.05, *p* < 0.001, *p* < 0.0001 (*, **, ***; respectively).

## Results

### Cortical Lesion Increases Brain Resident Immune and Peripheral Infiltrating Cells

**Figure 2:**
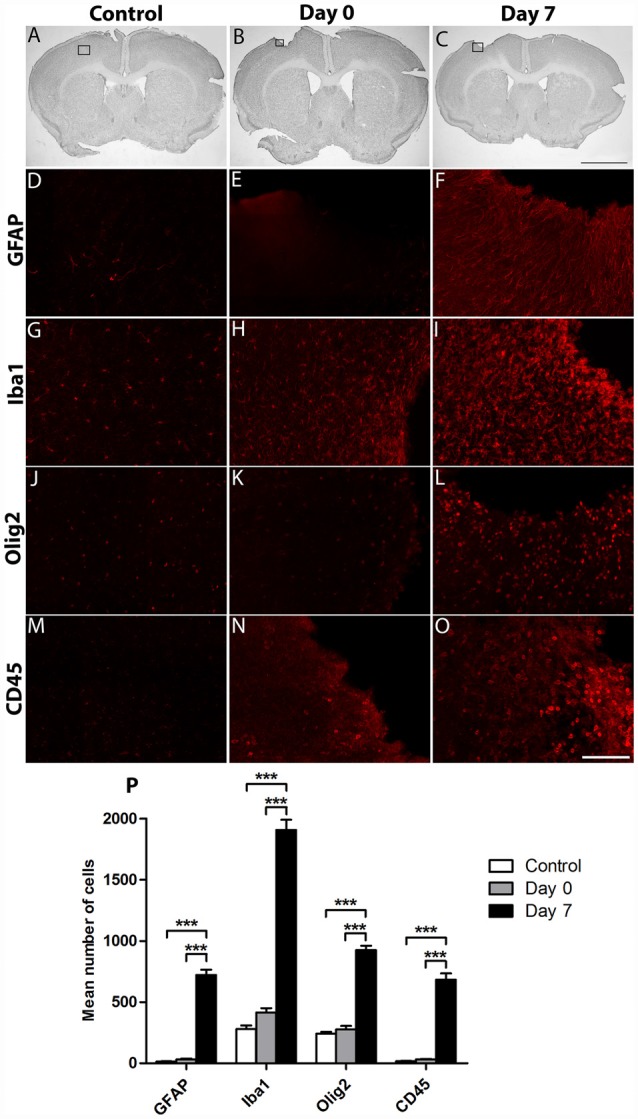
Characterization of the environment surrounding cortical lesion. The effect of cortical lesion on the number of resident immune cells and peripheral infiltrating cells in the injured cortex was evaluated in three groups of mice: control without lesion, Day 0 of lesion or Day 7 after lesion. Black squares show area of interest in Control **(A)**, Day 0 **(B)** or Day 7 **(C)** groups. Immunostaining using antibodies against Glial fibrillary acidic protein (GFAP; **D–F**), Iba1 **(G–I)**, Olig2 **(J–L)** or CD45 **(M–O)** allowed the detection of astrocytes, microglia, oligodendrocytes or hematopoietic cells in control, day 0, and day 7 after cortical lesion; respectively. **(P)** Histogram showing the quantification of the number of Iba1+, GFAP+, Olig2+ or CD45+ cells in different groups. The *p*-value was determined using two-way analysis of variance (ANOVA) followed by Bonferroni test. Values for ***p < 0.0001 were considered significant. Results are expressed as mean ± SEM. Results are representatives of *n* = 6 animals per group. Scale bar: **(A,B)** 2 mm **(D–O)** 80 μm.

To examine the effects of cortical lesion on the number of resident immune cells and peripheral infiltrating cells, IHC was used to identify GFAP+ astrocytes, Iba1+ microglial cells/macrophages, Olig2+ oligodendrocytes, and CD45+ hematopoietic cells (Figures 2A–C). As expected, the basal number of GFAP+ (13 ± 2; Figures 2D,P); Iba1+ (279 ± 29; Figures 2G,P); Olig2+ (240 ± 17; Figures 2J,P) and CD45+ (17 ± 2; Figures 2M,P) cells was low in the cortex of control group. At the day of lesion (day 0), no significant change of the number of cells was observed, in comparison to controls (GFAP: 31 ± 6; Iba1: 415 ± 34; Olig2: 277 ± 28; CD45: 32 ± 3; Figures 2E,H,K,N,P). However, at day 7 after the lesion, the number of astrocytes (721 ± 43), microglia (1,907 ± 82), oligodendrocytes (925 ± 36) and hematopoietic cells (683 ± 51) was significantly increased, in comparison to control and day 0 groups (***, *p* < 0.0001; Figures 2F,I,L,O,P). In addition, astrocytes and microglia showed morphological changes; indeed, astrocytes became hypertrophic whereas microglia presented an ameboid morphology (Figures 2F,I). Thus, a delay of 1-week after cortical lesion results in the recruitment and activation of inflammatory brain resident mediators and peripheral infiltrating cells.

### Differential Expression Kinetics of IL-1β and TGF-β1 Following Cortical Lesion

To evaluate the level of neuroinflammation following cortical lesion, using ISH, we investigated the spatiotemporal mRNA expression profile of pro- and anti-inflammatory cytokines, Interleukin-1 β (IL-1β) and TGF-β1; respectively. Both cytokines exhibited a differential expression at the lesion site, whereas they were neither present in the contralateral side nor in the cortex adjacent to the lesion site (data not shown).

**Figure 3:**
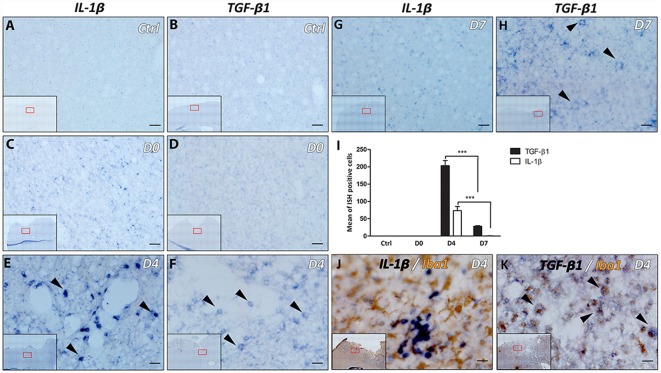
Differential expression of IL-1β and transforming growth factor-β1 (TGF-β1) following lesion of the motor cortex. The presence of IL-1β and TGF-β1 mRNAs was tested in the cortex without lesion (Ctrl; **A,B**) at the day of lesion (D0; **C,D**), 4 days (D4; **E,F**) and 7 days (D7; **G,H**) after the lesion (in blue, black arrowheads). Quantification analysis of the mean number of IL-1β and TGF-β1-expressing cells, in Ctrl, D0, D4 and D7 groups **(I)**. Co-labeling of IL-1β **(J)** or TGF-β1 **(K)** transcripts (in blue) with Iba1 macrophage/microglia marker (in brown) 4 days after lesion (black arrowheads). Red squares in inserts show area of interest. Data are presented as group mean ± SEM and asterisk indicates statistically significant differences (Student’s *T*-test, ***p < 0.0001). Scale bar: 20 μm.

Expression of IL-1β and TGF-β1 was not detected in the cortex of control animals with no lesion or in the cortex at day 0 of the lesion (Figures 3A–D,I). However, a strong expression of IL-1β was observed 4 days after lesion, within the cortex around the lesion cavity (Figures 3E,I), which disappeared at 1-week after the lesion (Figures 3G,I). On the other hand, TGF-β1 expression was strongly concentrated in the vicinity of the cortical lesion and to a lesser extent in the corpus callosum adjacent to the lesion site, 4 days after injury (Figures 3F,I). However, expression of TGF-β1 decreased significantly, but was still present, at 1-week after the lesion (Figures 3H,I).

Furthermore, the microglial/macrophagic phenotype (Iba1+) of the cells expressing IL-1β and TGF-β1 was analyzed 4 days after the lesion, using IHC in combination with ISH. Results showed that only TGF-β1+ cells expressed Iba1 marker (Figures 3J,K). On the other hand, IL-1α, IL-4, IL-6, IL-10, TNFα and LIF transcripts were not detected at day 0, day 4 or day 7 time points (data not shown).

### Temporal Kinetics of Microglia/Macrophage Polarization After Cortical Lesion

**Figure 4:**
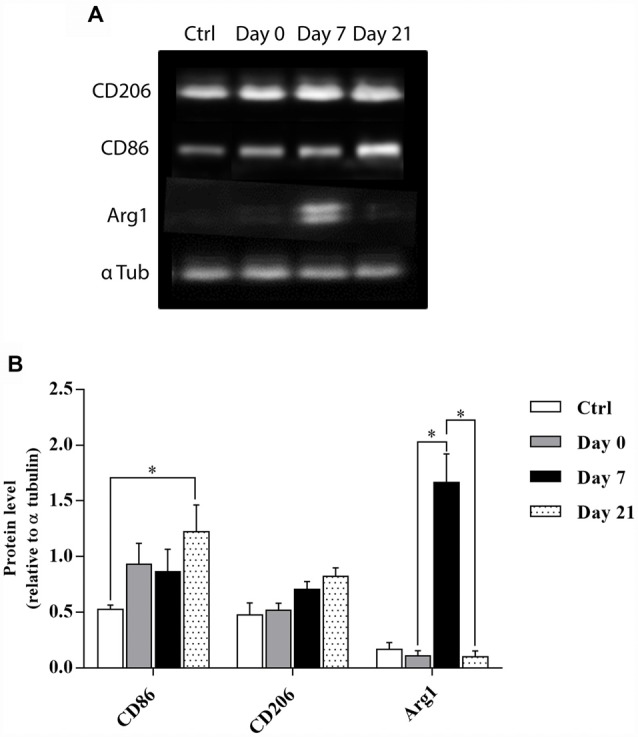
Temporal kinetics of microglia/macrophage polarization after cortical lesion. Western blot analysis for CD86, CD206, and Arg1 protein expression from the cell lysate of control and lesioned motor cortex sacrificed at different time points: day 0, day 7 and day 21 **(A)**. Quantification analysis of CD86, CD206, and Arg1 expression by normalizing to α tubulin level in ctrl, day 0, day 7, and day 21 groups **(B)**. The *p*-value was determined using Kruskal-Wallis test followed by Dunn’s multiple comparisons test. Values for *p < 0.05 were considered significant. Results are expressed as mean ± SEM. Results are representatives of *n* = 4 animals per group.

In order to further clarify whether the activated microglia/macrophages observed after cortical lesion were neurotoxic or neuroprotective, temporal changes in the phenotypes of M1- or M2- associated proteins (CD86 vs. CD206 and Arg1, respectively) were investigated in the motor cortex of control, day 0, day 7, and day 21 lesioned groups (Figure 4A). Quantitative analysis showed that the expression of M1 marker CD86 slightly increased at day 0 and day 7, while a significant increase was observed at day 21 after injury. In contrast, the expression of M2 marker Arg1 peaked at 1-week after the lesion and returned to pre-injury levels at day 21 (Figure 4B) whereas M2 marker CD206 showed no significant difference between the 4 groups. Our results demonstrated that an M1 microglia phenotype is dominant in the vicinity of the lesion. However, an M1-to-M2 switch was observed at 1-week after the lesion followed by a replacement of the transient M2 response by an M1 response at day 21 post-lesion.

### Cortical Transplantation Modified Brain Resident Immune and Peripheral Infiltrating Cells

**Figure 5:**
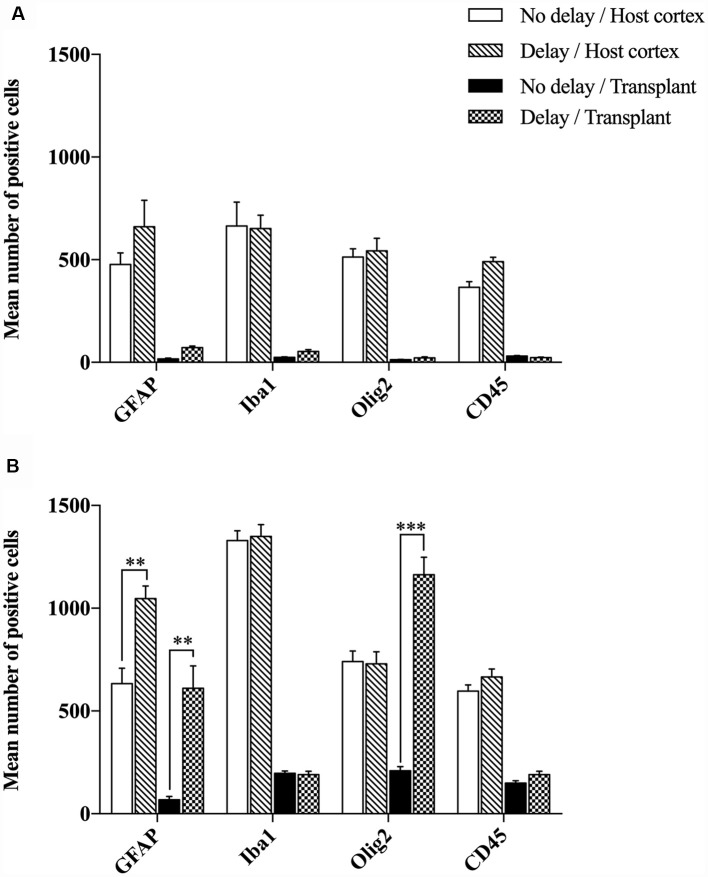
Histogram showing quantifications of GFAP+, Iba1+, Olig2+ or CD45+ cells at 4 days **(A)** and 7 days **(B)** after cortical transplantation. Results are expressed as mean ± SEM. Results are representatives of *n* = 6 animals per group. The *p*-value was determined using Student’s *T*-test. Values for ***p < 0.0001 and **p < 0.001 were considered significant.

The effects of a 1-week delay or no delay, between lesion and transplantation on inflammatory responses were examined at 4- or 7-days post-transplantation, in the graft and the cortical surrounding areas. Four days after transplantation, the number of brain resident immune cells (Iba1+, GFAP+, Olig2+) and peripheral infiltrating cells (CD45+) were not significantly different between the two groups of transplanted animals, with or without delay, whether in the host cortex adjacent to the transplant (Iba1+, No delay: 665 ± 116; Delay: 652 ± 65; GFAP+, No delay: 477 ± 56; Delay: 661 ± 128; Olig2+, No delay: 513 ± 41; Delay: 543 ± 61; CD45+, No delay: 365 ± 27; Delay: 491 ± 20) or within the transplant (Iba1+, No delay: 24 ± 2; Delay: 53 ± 9; GFAP+, No delay: 17 ± 4; Delay: 71 ± 8; Olig2, No delay: 13 ± 2; Delay: 22 ± 5; CD45+, No delay: 30 ± 3; Delay: 24 ± 2; Figure 5A).

**Figure 6:**
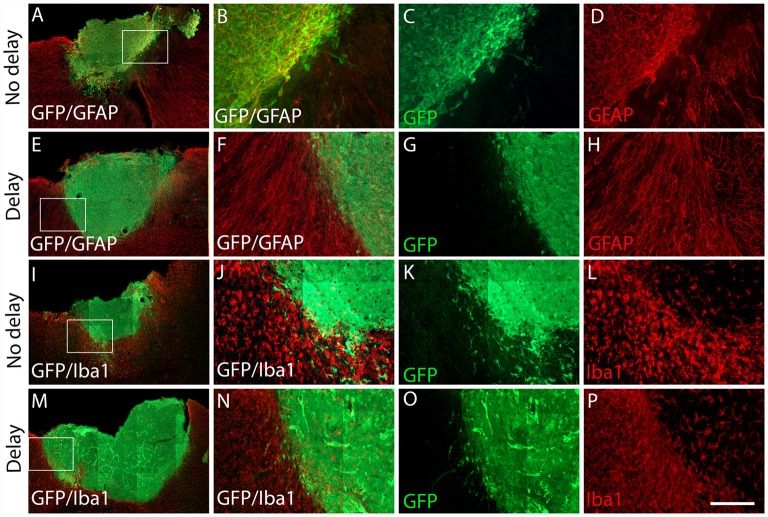
Expression of astrocytes and microglia at 7 days after cortical transplantation. Low magnification photomicrographs of coronal sections illustrating the immunolabeled immune cells (red) in the GFP+ transplants (green) at day 7 after transplantation **(A,E,I,M)**. Astrocytes **(A,E)** and microglia **(I,M)** after transplantation with no delay **(A,I)** or with delay of 1-week **(E,M)** after the cortical lesion. High magnification images from regions of interest showing immunolabeled astrocytes **(B–D,F–H)**, microglia **(J–L,N–P)**, in the GFP+ transplants (in green) after transplantation with no delay **(B–D,J–L)** or with delay **(F–H,N–P)** of 1-week after the cortical lesion. Scale bars: **(A,E,I,M)** 480 μm, **(B–D,F–H,J–L,N–P)** 80 μm.

**Figure 7:**
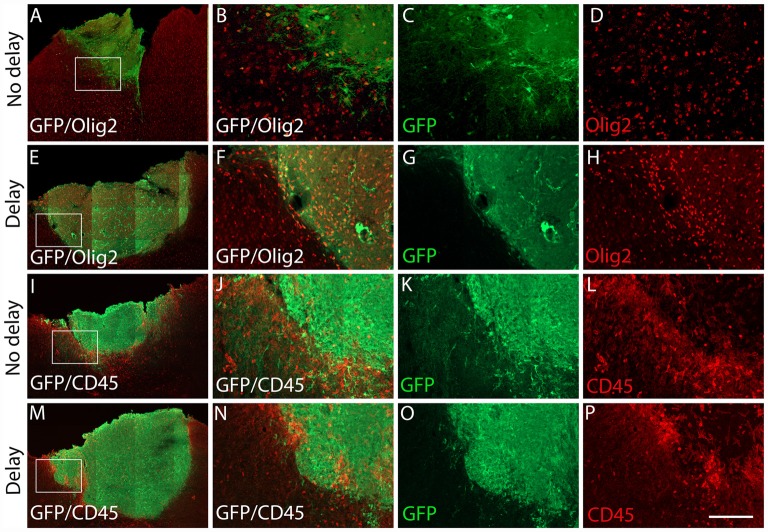
Expression of oligodendrocytes and hematopoietic cells after cortical transplantation. Low magnification photomicrographs of coronal sections illustrating the immunolabeled immune cells (red) in the GFP+ transplants (green) at day 7 after transplantation **(A,E,I,M)**. Oligodendrocytes **(A,E)** and hematopoietic cells **(I,M)** after transplantation with no delay **(A,I)** or with delay of 1-week **(E,M)** after the cortical lesion. High magnification images from regions of interest showing immunolabeled oligodendrocytes **(B–D,F–H)** and hematopoietic cells **(J–L,N–P)** in the GFP+ transplants (in green) after transplantation with no delay **(B–D,J–L)** or with delay of 1-week **(F–H,N–P)** after the cortical lesion. Scale bars: **(A,E,I,M)** 480 μm, **(B–D,F–H,J–L,N–P)** 80 μm.

Seven days after transplantation, the number of microglial (Iba1+) and hematopoietic (CD45+) cells were not significantly different between the two groups of transplanted animals, with or without delay, whether in the host cortex adjacent to the transplant (Iba1+, No delay: 1,329 ± 57; Delay: 1349 ± 47; Figures 6I–P, 5B; CD45+, No delay: 666 ± 38; Delay: 596 ± 30; Figures 7I–P, 5B) or within the transplant (Iba1+, No delay: 196 ± 15; Delay: 191 ± 11; Figures 6I–P, 5B), (CD45+, No delay: 191 ± 15; Delay: 149 ± 11; Figures 7I–P, 5B). However, a significant increase in the number of astrocytes (GFAP+ cells) was observed in the host cortex in the group of animals transplanted with a delay of 1-week, compared to the no delay group (GFAP+, No delay: 632 ± 75; Delay: 1,047 ± 61; Figures 6A–H, 5B). Similarly, a significant increase in astrocytes was also detected in the transplant in the delay group (No delay: 68 ± 16; Delay: 610 ± 109; Figures 6A–H, 5B). On the other hand, while numerous oligodendrocytes (Olig2+ cells) were detected in the host cortex, no significant difference was found between the two groups (Olig2+, No delay: 740 ± 50; Delay: 729 ± 58; Figures 7A–H, 5B). However, a significant increase was observed in the transplant for the group with a delay of 1-week, in comparison to that with no delay (Olig2, No delay: 209 ± 20; Delay: 1,163 ± 84; Figures 7A–H, 5B). In addition, astrocytes and microglia showed morphological changes. Indeed, astrocytes presented different morphologies depending on their distance from the transplant. In fact, astrocytes were more elongated and showed “palisading” morphology in the vicinity of the transplant. Furthermore, hypertrophic astrocytes were orientated towards the injury site, whereas GFAP+ astrocytes were present but showed no signs of hypertrophy at the border of the unaffected corpus callosum. Palisading astrocytes were more observed in the host cortex in the no delay group in comparison to the delay group (Figures 6A,E). However, microglia were more ameboid/less ramified in the no delay group compared to the delay group (Figures 6I,M). Our results showed that a 1-week delay between lesion and transplantation enhanced the number of astrocytes in the host adjacent cortex as well as in the transplant, whereas oligodendrocytes increased only within the transplant.

### Astrocytes and Microglia/Macrophage Polarization After Cortical Transplantation

**Figure 8:**
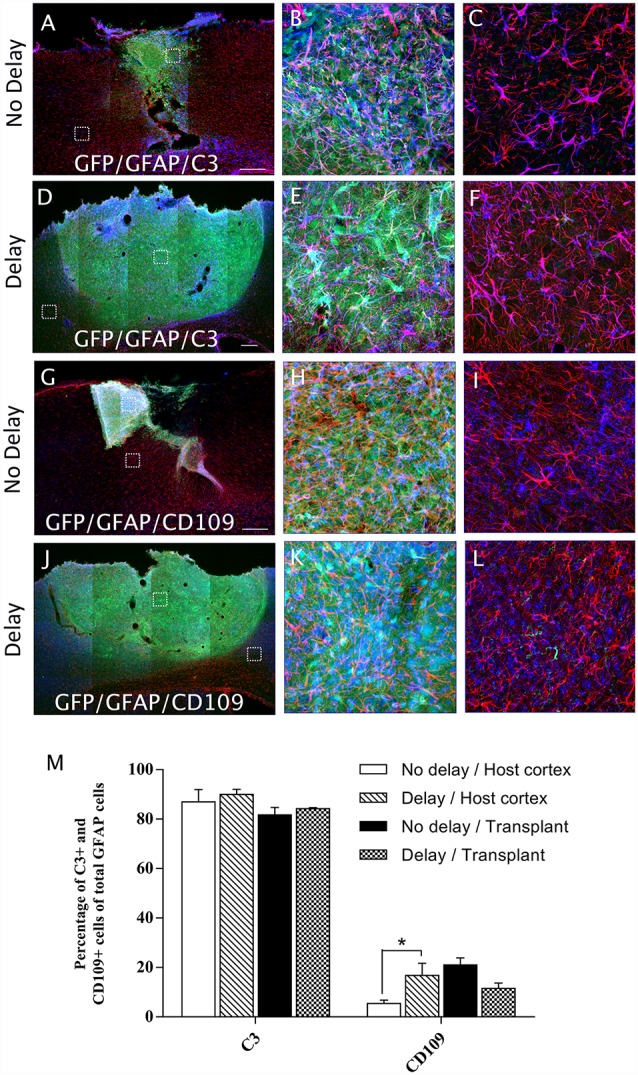
Astrocytes polarization after cortical transplantation. Low magnification photomicrographs of coronal sections illustrating the astrocytes (red) and their phenotype (blue) in the GFP+ transplants (green) at day 7 after transplantation **(A,D,G,J)**. A1 astrocytes (GFAP+/C3+; **A,D**) and A2 astrocytes (GFAP+/CD109+; **G,J**) after transplantation with no delay **(A,G)** or with 1-week delay **(D,J)** after the cortical lesion. High magnification images from regions of interest showing GFAP+/C3 cells **(B,C,E,F)**, GFAP+/CD109 cells **(H,I,K,L)**, in the GFP+ transplants (in green) after transplantation with no delay **(B,C,H,I)** or with delay **(E,F,K,L)** of 1-week after the cortical lesion. Scale bars: **(A,D,G,J)** 480 μm. Histogram showing quantifications of the percentage of C3+ or CD109+ cells of total GFAP+ cells. **(M)** Results are expressed as mean ± SEM. The *p*-value was determined using Kruskal-Wallis test and Dunn’s multiple comparison *post hoc* test, *p < 0.05.

**Figure 9:**
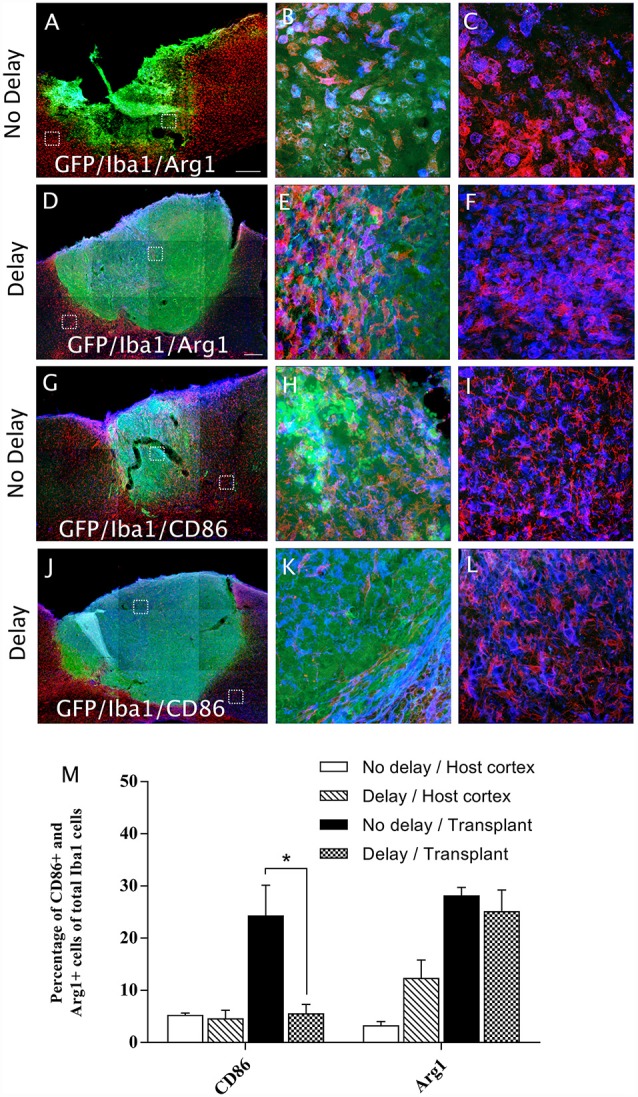
Microglia/macrophage polarization after cortical transplantation. Low magnification photomicrographs of coronal sections illustrating the microglia cells (red) and their phenotype (blue) in the GFP+ transplants (green) at day 7 after transplantation **(A,D,G,J)**. M2 microglia phenotype (Iba1+/Arg1+; **A,D)** and M1 microglia phenotype, (Iba1+/CD86+; **G,J**) after transplantation with no delay **(A,G)** or with delay of 1-week **(D,J)** after the cortical lesion. High magnification images from regions of interest showing Iba1+/Arg1+ cells **(B,C,E,F)**, Iba1+/CD86+ cells **(H,I,K,L)**, in the GFP+ transplants (in green) after transplantation with no delay **(B,C,H,I)** or with delay **(E,F,K,L)** of 1-week after the cortical lesion. Scale bars: **(A,D,G,J)** 480 μm. Histogram showing quantifications of the percentage of Arg1+ or CD86+ cells of total Iba1+ cells. **(M)** Results are expressed as mean ± SEM. The *p*-value was determined using Kruskal-Wallis test and Dunn’s multiple comparison *post hoc* test, *p < 0.05.

In order to examine the pro-inflammatory vs. anti-inflammatory profile of microglia/macrophage and astrocytes, IHC was used to identify astrocytes and microglia/macrophage subpopulation in the graft and the cortical surrounding areas at 7-days post-transplantation. While numerous A1 astrocytes (C3+ cells) were detected in the host cortex and transplant, no significant difference was found between the two groups (Host cortex, No delay: 87 ± 4.96%; Delay: 89.93 ± 2.15%; Transplant, No delay: 81.72 ± 3%; Delay: 84.2 ± 0.43%; Figures 8A–F,M). In contrast, a significant increase in the percentage of A2 astrocytes (CD109+ cells) was observed in the host cortex for the group with a 1-week delay, in comparison to that with no delay (No delay: 5.43 ± 1.4%; Delay: 16.8 ± 4.9%; Figures 8G–M). Regarding microglia/macrophage polarization, we performed double labeling of Iba1 with arginase-1 (Arg1, M2 phenotype) and showed that the percentage of Iba1+/Arg1+ cells was not significantly different between the two groups of transplanted animals, with or without delay, whether in the host cortex adjacent to the transplant (No delay: 2.50 ± 0.17%; Delay: 12.27 ± 3.53%; Figures 9A,C,D,F,M) or within the transplant (No delay: 28.08 ± 1.66%; Delay: 25.08 ± 4.15%; Figures 9A,B,D,E,M). In addition, we performed double labeling of Iba1 with M1 phenotype marker, CD86, and showed no significant difference in the percentage of Iba+/CD86+ cells in the host cortex adjacent to the transplant in the two groups of transplanted animals (No delay: 5.19 ± 0.46%; Delay: 4.52 ± 1.65%; Figures 9G,I,J,L,M). Substantially, more Iba1+ cells expressed the M1 marker CD86 in the transplant of animals transplanted without a delay, compared to the delay group (No delay: 24.21 ± 5.95%; Delay: 5.47 ± 1.85%; Figures 9G,H,J,K,M). Our results showed that a 1-week delay between lesion and transplantation increased the percentage of A2 astrocytes in the host adjacent cortex, whereas M1 microglia decreased only within the transplant.

## Discussion

We have recently shown that a time delay of 1-week between a lesion in the adult mouse motor cortex and homotopic cortical transplantation of embryonic cells significantly enhance vascularization, proliferation, and survival of grafted cells as well as density projections developed by grafted neurons. Moreover, we have also shown that this delay has beneficial impacts on functional repair and recovery (Péron et al., 2017). The mechanisms leading to these positive outcomes have not been yet defined. It has been postulated that potential benefits of introducing a delay between the lesion and transplantation may result from the release of trophic factors secreted by cells surrounding the lesion (Nieto-Sampedro et al., 1983), the secretion of pro-angiogenic factors (Sköld et al., 2005; Dray et al., 2009), the decrease of toxin levels (Gonzalez and Sharp, 1987) or the modulation of inflammation levels (Zhang et al., 2010; Burda et al., 2016). Thus, the present study was designed to determine the effect of a 1-week delay on post-traumatic inflammation.

Here, we showed morphological changes in astrocytes and microglia, but also an increase in the number of astrocytes, microglia, oligodendrocytes and CD45+ cells 7 days after the lesion, in comparison to lesion at day 0. In accordance with our results, many studies have shown that microglia responds rapidly to CNS injuries, becoming hypertrophic and reaching its maximum level within the first week after cortical lesion before gradually disappearing (Davalos et al., 2005; Ladeby et al., 2005; Turtzo et al., 2014). In addition, the blood-brain barrier is damaged after the lesion allowing circulating macrophage cells to infiltrate into the site of injury (Shlosberg et al., 2010). In parallel, it has been shown that within 24 h after injury astrocytes proliferate, increase their expression of GFAP, and become hypertrophic (Raivich et al., 1999; Burda et al., 2016) as demonstrated in this study. Following a cortical stab injury, GFAP+ cells were absent at day 2 and 4 post-lesion in the area close to the lesion whereas large numbers of hypertrophic astrocytes were revealed at day 7, demonstrating that most of the increase in GFAP+ cells near the lesion is not due to cell division but is caused by process extension or migration (Hampton et al., 2004). Furthermore, a strong astrogliosis was identified from 7 days until 2 months after injury, where astrocytes contributed to the formation of the glial scar (Villapol et al., 2014).

While activated microglial and macrophage cells express pro- and anti-inflammatory cytokines, we found that the expression of pro-inflammatory cytokine IL-1β starts 1 day after the lesion (data not shown), increased at day 4 and became undetectable at 1-week. In addition, anti-inflammatory cytokine TGF-β1 was strongly expressed at 4 days after the lesion, which decreased significantly at 7 days, but was still detectable. Interestingly, 19% of TGF-β1+ cells co-expressed Iba1, which supports the previous hypothesis that microglial/macrophages activation is also neuroprotective (Lai and Todd, 2006). Based on our previous work showing that a delay of 1-week between cortical lesion and transplantation leads to a transient but significant increase in graft vascularization (Péron et al., 2017) along with the knowledge that TGF-β1 is pro-angiogenic *in vivo* and induces angiogenesis (Roberts et al., 1986; Madri et al., 1988; Yang and Moses, 1990; Evrard et al., 2012), we suggest that the increase in graft vascularization observed in the delay group could be in part due to the increased expression of TGF-β1.

Multiple reports on microglia in the injured CNS provide strong support for dual microglial roles, both beneficial and deleterious, as well as differential microglial activation into M1 (pro-inflammatory) or M2 (anti-inflammatory) phenotypes (Kigerl et al., 2009; Perry et al., 2010). In other words, microglia can either promote delayed cell damage by generating pro-inflammatory cytokines and oxidative stress or participate in regenerative processes by clearing debris *via* phagocytosis and release of trophic factors (Hu et al., 2015). In order to further clarify the role of activated microglia/macrophages observed after cortical lesion, M1 and M2 phenotypes were discriminated through further analyses. M1 phenotype was found to dominate the cortical lesion site immediately after the lesion (at day 0) while an M1-to-M2 switch was observed at 1-week after lesion, demonstrated by the expression of Arg1. However, the transient M2 response was replaced by a chronic M1 response (M2-to-M1 switch) at 21 days after lesion. Similar switches have already been reported in models of spinal cord injury (SCI) where an M2 macrophage response was observed depleting within 3–7 days after SCI and which returned to baseline at 14 days (Kigerl et al., 2009; David and Kroner, 2011). In addition, an alternatively activated subset of M2 microglia/macrophage occupied the site of lesion at day 5 after and phased out within 7 days followed by a shift to M1 phenotype (Wang et al., 2013) until 28 days post-injury (Jin et al., 2012). Interestingly, studies of microglia and macrophages after demyelinating lesion revealed an M1-to-M2 switch at the initiation of remyelination, identifying the regenerative capacity of M2 cells in the CNS (Miron et al., 2013).

It is likely that M1 and M2 exist in a state of dynamic equilibrium within the acute lesion microenvironment in the brain. Neuroinflammation is initially a protective response, however, excess inflammatory responses are detrimental, and they may inhibit neuronal regeneration (Russo and McGavern, 2016). Previous studies suggested that expression of M1 microglia/macrophages impair axonal regrowth (David and Kroner, 2011) in contrast to the expression of M2 microglial factors that promote CNS repair while limiting secondary inflammation in the context of neuronal injury (Kigerl et al., 2009). Therefore, M1/M2 switching may help therapeutic effectiveness in traumatic injuries, ischemic damages, and neurodegenerative diseases (Le et al., 2016; Orihuela et al., 2016). It is thus conceivable that the M1-to-M2 shift observed in our model at 1-week post-lesion, in comparison to day 0 and day 21, could enhance clearance of necrotic debris without causing toxicity while also promoting the outgrowth of grafted neurons. Therefore, the polarized M2 microglia/macrophages response observed nearby the lesion at 1-week post-injury, but not immediately after the lesion (day 0), may represent an endogenous effort to restrict brain damage and might be implicated in the ameliorations that we observed previously when transplanting with time delay (Péron et al., 2017).

The influence of transplanted cells was then studied on the modulation of inflammatory response in the host after transplantation, with or without a delay of 1 week. Seven days post-transplantation, we observed an increase in the number of astrocytes in the host cortex adjacent to the transplant and in the transplant. In addition, despite a high percentage of A1 astrocyte phenotype in the cortex and the transplant in grafted animals with or without delay, no significant differences were observed between groups. In contrast, a significant increase in the percentage of A2 astrocyte phenotype was observed in the host cortex of the delay group in comparison to no delay group. It has been shown that host astrocytes re-expressed developmental factors to direct axonal growth of the transplant (Gaillard and Jaber, 2007). In addition, astrocytes presented an early phenotype that can partially cause the active migration of transplanted neurons and neuronal precursors (Leavitt et al., 1999). Previous studies have suggested that the main function of reactive astrocytes is to create a physical barrier between damaged and healthy cells (Silver and Miller, 2004), repair the blood-brain barrier (Faulkner et al., 2004), and reduce the excess of glutamate (Zou et al., 2010). Astrocytes can also secrete different growth factors such as BDNF (Schwartz et al., 1994) and VEGF (Rosenstein and Krum, 2004). Astrocytes, as a result of their close relationships with neurons, microglia, and blood vessels have long been hypothesized to be involved in cerebrovascular regulation (Sochocka et al., 2013). In line with our study, it has been demonstrated that astrocytes, starting at 7 days after injury, establish a link with large vessels at the border of the lesion site (Villapol et al., 2014). In summary, we suggest that the expression of trophic factors by astrocytes after transplantation creates a more favorable environment for the development of the transplant. On the other hand, the 1-week delay did not show any effect on the expression of microglia and hematopoietic cells in the transplanted groups. Conversely, the number of microglia decreased after transplantation, which suggests that microglia would act mainly to remove cell debris and promote tissue integration 1-week after the lesion. Among microglia, we have observed a higher percentage of Iba1+ cells expressing proinflammatory M1 marker CD86 in the transplant of the no delay group in comparison to the delay group. These observations suggest that, in no delay transplantation group, host environment induced M1 polarization of microglia within the graft which could be deleterious for the grafted neurons. Indeed, M1 activation in the brain can induce neurotoxicity due to the release of pro-inflammatory factors and neurotoxic mediators (Gao et al., 2003; Qin et al., 2004).

In a previous study, we grafted embryonic motor cortical neurons into the adult mouse motor cortex immediately after the lesion and reported that 30% of the axons derived grafted neurons were myelinated (Gaillard et al., 2007). Interestingly, in the present study, the number of oligodendrocytes was higher in the transplant grafted with a delay compared to no delay, suggesting a more favorable myelination of transplanted neuronal axons with delay.

## Conclusion

The introduction of a delay of 1-week between the cortical lesion and transplantation of embryonic neurons is beneficial at the neuroanatomical level and functional recovery. Our data on a delay of 1-week resulted into: (1) an increase of inflammation levels; (2) presence of anti-inflammatory cytokine TGF-β1 and absence of pro-inflammatory cytokine IL-1β; and (3) domination of M2 phenotype response. We have also shown an increased expression of GFAP+ cells after transplantation with delay. These results suggested that neuroinflammation observed 1-week after cortical lesion might be promoting debris clearance, graft vascularization, myelination of transplanted neurons axons, and development of projections by grafted neurons. Thus, the environment surrounding cortical lesion could be favorable to the development of transplanted neurons. A detailed analysis of toxins, trophic, and pro-angiogenic factors and the time course of their release is necessary to determine their implication in the benefits shown when transplanted with a 1-week delay. It is also important to identify the molecular and cellular cues that drive microglia and macrophages toward an M2 phenotype after cortical lesion. By doing so, the inflammatory response could be shifted away from the harmful M1 phenotype and complementary therapeutic targets could be revealed.

Currently, various sources of cells are investigated for cell-based therapies. Fetal tissue transplants represent a promising strategy in cell-based regenerative medicine; however, their use is ethically restricted which limit the development of such an approach (Lindvall et al., 2004). To overcome this limitation, cortical neurons derived from embryonic stem cells (ESCs) or induced pluripotent stem cells (iPSCs) represent a promising alternative cell source for cell therapy (Tornero et al., 2013; Dunkerson et al., 2014). Indeed, several studies reported that mouse (Gaspard et al., 2008; Michelsen et al., 2015) and human (Espuny-Camacho et al., 2013, 2018) ESCs can be differentiated into cortical neurons. Cortical neurons derived stem cells grafted into the cortex of newborn (Gaspard et al., 2008; Espuny-Camacho et al., 2013) or adult mice (Michelsen et al., 2015; Espuny-Camacho et al., 2018) send long-distance projections to appropriate cortical and subcortical targets. Extensive investigations are necessary to secure motor cortical identity/fate of the neurons derived from pluripotent stem cells for successful transplantation.

## Ethics Statement

All animal experimentation and housing were carried out in accordance with the guidelines of the French Agriculture and Forestry Ministry (decree 87849) and the European Communities Council Directive (2010/63/EU). All experiments were conducted in compliance with current Good Clinical Practice standards and in accordance with relevant guidelines and regulations and the principles set forth under the Declaration of Helsinki (1989). All efforts were made to reduce the number of animals used and their suffering.

## Author Contributions

AG conceived the idea and supervised the whole project. NB performed the majority of the experiments with the help of SB, LP, TR, M-LB and MF. NB analyzed the data. AG, NB, KZ, SB and LP interpreted the data and wrote the article.

## Conflict of Interest Statement

The authors declare that the research was conducted in the absence of any commercial or financial relationships that could be construed as a potential conflict of interest.

## Abbreviations

BDNF: brain-derived neurotrophic factor
CNS: central nervous system
DAPI: 4′,6-diamidino-2-phenylindole
GDNF: Glial cell-line derived neurotrophic factor
GFAP: Glial fibrillary acidic protein
GFP: green fluorescent protein
IHC: immunohistochemistry
IL: interleukin
ISH: *in situ* hybridization
LIF: leukemia inhibitory factor
LPS: Lipopolysaccharide
NGF: nerve growth factor
PBS: phosphate-buffered saline
PCR: polymerase chain reaction
TGF-β1: transforming growth factor-β1
TNFα: tumor necrosis factor α.
